# Combination of resveratrol and 5-flurouracil enhanced anti-telomerase activity and apoptosis by inhibiting STAT3 and Akt signaling pathways in human colorectal cancer cells

**DOI:** 10.18632/oncotarget.25993

**Published:** 2018-08-31

**Authors:** Seyung S. Chung, Pranabananda Dutta, David Austin, Piwen Wang, Adam Awad, Jaydutt V. Vadgama

**Affiliations:** ^1^ Division of Cancer Research and Training, Department of Internal Medicine, Charles R. Drew University of Medicine and Science, Los Angeles, CA, USA; ^2^ Jonsson Comprehensive Cancer Center, University of California at Los Angeles, Los Angeles, CA, USA; ^3^ David Geffen School of Medicine, University of California at Los Angeles, Los Angeles, CA, USA

**Keywords:** resveratrol, telomerase, combination treatment, colorectal cancer, STAT3

## Abstract

Colorectal cancer is one of the leading causes for mortalities worldwide. The most common cause of colorectal cancer mortality is hepatic metastasis. There has been a limited advancement in the targeted-therapies for metastatic colorectal cancer. Conventional chemotherapeutic agent 5-fluorouracil has been used for various cancer treatments including colorectal cancer. Development of drug resistance and severe toxicity are major hurdles for its use in clinical setting. Resveratrol is a natural polyphenolic compound which has protective effects against aging-related diseases. In this study, we have tested whether combined treatments of resveratrol and 5-FU enhanced inhibitory effects against colorectal cancer cell growth. We herein showed that resveratrol and 5-FU combination treatments caused the anti-cancer activities by simultaneously inhibiting STAT3 and Akt signaling pathways. Resveratrol treatment induced S-phase specific cell cycle arrest, and when combined with 5-FU, it showed further increase in colorectal cancer cell apoptosis. Combined treatments of resveratrol and 5-FU inhibited epithelial-mesenchymal transition. Notably, resveratrol showed anti-inflammatory effects by downregulating inflammatory biomarkers, pSTAT3 and pNFκB. Resveratrol and 5-FU treatments inhibited STAT3 phosphorylation and its binding to the promoter region of human telomerase reverse transcriptase (hTERT). Our data provide the first evidence that resveratrol can enhance anti-telomeric and pro-apoptotic potentials of 5-FU in colorectal cancer, hence lead to re-sensitization to chemotherapy.

## INTRODUCTION

Colorectal cancer is the third most prevalent type of cancer and second leading cause of cancer-related death worldwide [1]. The leading cause of death from colorectal cancer is hepatic metastasis [2]. More than 50% of colorectal cancer patients progress toward metastases during the treatment or at the recurrence [3]. Colorectal cancer is primarily treated with surgical removal of the tumor, conventional chemotherapy, and with or without radiation [4]. When detected at advanced stages, colorectal cancer is difficult to treat or cure the disease. Despite multiple new FDA approved therapies, 5-year survival rate is lower than 10% in advanced colorectal cancer patients [5]. 5-Fluorouracil (5-FU) has been used for the treatment of various solid tumors including gastrointestinal cancers [6]. It is a drug that inhibits enzyme activity of thymidylate synthase during DNA replication. 5-FU induces the G1/S cell cycle arrest that leads to apoptosis functioning as an antimetabolite [7]. Adjuvant chemotherapy with 5-FU, leucovorin and oxaliplatin (FOLFOX) is routinely recommended for stage II and III colon cancer patients [8]. However, patients frequently develop drug resistance against chemotherapy and suffer from severe side-effects including liver damage [9]. Although the treatment may seem to be temporarily useful, adjuvant chemotherapy rarely cures colorectal cancer and the disease relapses over time [10]. Therefore, there is a significant need for developing new treatments that include novel targets and are less toxic.

Resveratrol is a naturally occurring phytoalexin found in various plants including grapes, nuts and berries and is frequently produced in response to stress, injury or fungal infection [11]. In addition, resveratrol exerts multiple bioactivities of antioxidants, anti-aging, insulin sensitization and reduction of risks for cardiovascular disease [12]. Resveratrol interacts with various molecular targets associated with inflammation and immunity [13]. Protective nature of resveratrol may hold clinical importance as an anti-inflammatory drug. Increasing evidences suggest the anti-tumor effects of resveratrol during several processes during cancer initiation, angiogenesis, apoptosis and metastasis [14]. The anti-cancer mechanisms of resveratrol have been suggested during cell-cycle arrest, apoptosis, and in response to DNA damage [15]. Resveratrol was also reported to be pro-apoptotic via plasma membrane integrin αVβ3 signals transduced by extracellular-regulated kinases 1 and 2 in glioma cells [16]. In breast cancer cell line MCF7, integrin αVβ3 contained a receptor site for resveratrol which linked to p53 phosphorylation driven apoptosis [17]. To understand, the exact mechanisms of resveratrol as an anti-cancer agent need further studies. Interestingly, resveratrol has been shown to enhance the sensitization of radiotherapy and/or chemotherapy in bladder cancer and in intestinal adenoma [18, 19]. Considering its chemo sensitizing potential with a relatively low toxicity, we wished to test resveratrol as a chemosensitizing adjunct drug for the 5-FU chemotherapy.

Signal transducer and activator of transcription 3 (STAT3) is a latent transcription factor that conveys various growth signals from the cell membrane to the nucleus [20]. It is involved in many cellular processes including proliferation, survival, and immune responses [21]. In a variety of human malignancies, constitutive activation of STAT3 is correlated with tumor progression and poor prognosis [22]. Nuclear factor κB (NFκB) is a protein complex that regulates DNA synthesis and cell survival [23]. NFκB also plays a key role in immune response to infection [24]. Both STAT3 and NFκB are constitutively activated in many cancers [25]. In this study, we employed the phosphorylated STAT3 and NFκB as an inflammation biomarker in colorectal cancer and tested resveratrol for its anti-inflammatory effects.

Telomerase is composed of reverse transcriptase (TERT) and RNA component (TERC), and elongates the telomere DNAs at the end of chromosomes [26]. In most somatic cells, telomerase expression is transcriptionally repressed whereas in ~ 90 % of human malignancies, it is hyper-activated [27]. The simple and attractive cancer-specific characteristics of telomerase have been explored as a target for therapy in many cancers [28]. Considering the immortality of tumors, pursuing telomerase has the potential as an efficient, targeted-therapy from the deleterious responses in apoptosis and senescence against cancer. In addition to its role as a telomeric DNA polymerase, telomerase regulates a diverse array of physiological functions in oncogenesis [29]. It has been demonstrated that hTERT promoted the epithelial-mesenchymal transition and stemness in gastric cancer [30]. Recently, accumulating studies suggest that telomerase modulate not only telomeric elongation, but also regulate the cancer stem cell generation and maintenance through the cellular reprogramming processes [31]. This “non-canonical” function of telomerase has added its value as a target for therapy during the development of novel regimens for advanced colorectal cancer. Here, we hypothesized the combination treatments of a natural compound resveratrol and 5-FU might efficiently inhibit telomerase activity through the anti-inflammatory effects. We report the enhanced anti-telomerase caused by the inactivation of STAT3 and blocking of STAT3 binding to the hTERT promoter site upon the combined treatments. Moreover, we show that S-phase cell cycle arrest and increased apoptosis from the combination treatments. Proliferation and migration of colorectal cancer cells were also decreased with the treatments. Our results suggest that resveratrol and 5-FU combination can be an efficient therapeutic approach for the advanced colorectal cancer.

## RESULTS

### Resveratrol inhibited the colorectal cancer cell proliferation and, when combined with 5-FU, the cytotoxicity has been enhanced

**Figure 1:**
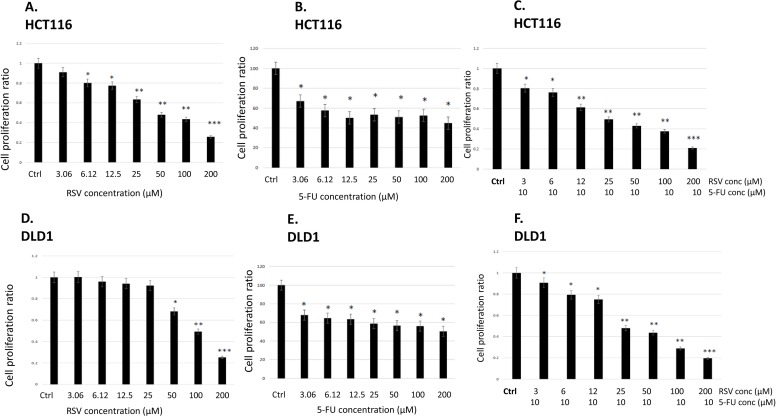
Effects of resveratrol and 5-FU on cell proliferation of human colorectal cancer cell lines HCT116 and DLD1 **(A)** Cell proliferation assay with resveratrol in HCT116. Cells were treated with resveratrol at 0 ~ 200 μM for 72 hours and applied to MTS assay. **(B)** HCT116 cells were treated with 5-FU at 0~200 μM for 72 hours. **(C)** HCT116 cells were treated with resveratrol at 0 ~ 200 μM combined with 5-FU at 10 μM for 72 hours and applied to MTS assay. **(D)** DLD1 cell proliferation assay with resveratrol at 0 ~ 200 μM for 72 hours. **(E)** DLD1 cells were treated with 5-FU at 0~ 200 μM for 72 hours. **(F)** DLD1 cells were treated with resveratrol combined with 5-FU at 10 μM for 72 hours. Error bars represent standard deviation.

First of all, we treated human colorectal cancer cell lines HCT116 and DLD1 with a concentration of resveratrol ranging from 0 to 200 μM for 72 hours and examined cell proliferation employing MTS assay as described in Materials and Methods (Figure 1A and 1D). IC_50_ of resveratrol was 42.5 μM when tested with HCT116, and IC_50_ of resveratrol was 100 μM in DLD1, respectively. In DLD1, resveratrol showed higher IC_50_ as the difference is possibly derived from the biology and genetic make-up of the cell line. HCT116 has a mutation in RAS oncogene while DLD1 has a mutation in p53 [32]. It has been reported that resveratrol exerted differential effects on proliferation of cancer cells from different origin which is mainly accompanied by p53 activation [33]. Secondly, we treated HCT116 and DLD1 with a concentration of 5-FU ranging from 0 to 200 μM for 72 hours and examined the cell proliferation (Figure 1B and 1E). Both cell lines showed drug resistance to 5-FU. We hypothesized that combined treatments with resveratrol and 5-FU might increase the sensitivity of colorectal cancer cells to 5-FU. Resveratrol has an anti-inflammatory effects and more importantly possesses chemo sensitizing ability for resistant cells to chemotherapeutic agents like 5-FU. Therefore, we have combined 10 μM of 5-FU with a gradient of resveratrol and repeated the cell proliferation assays. As shown in the figure, resveratrol cytotoxicity was enhanced further when combined with 5-FU both in HCT116 and DLD1. In HCT116, the IC_50_ of resveratrol is reduced from 42.5 μM to 26.6 μM while in DLD1, the IC_50_ has been decreased from 102 μM to 26 μM (Figure 1B and 1D). Our data showed that resveratrol has an anti-proliferative activity against colorectal cancer cells with a monotherapy and the cytotoxicity has been enhanced in combination with chemotherapy agent 5-FU in human colorectal cancer cells.

### Resveratrol and 5-FU treatments induced S phase specific cell cycle arrest with treatments alone and in combination

**Figure 2:**
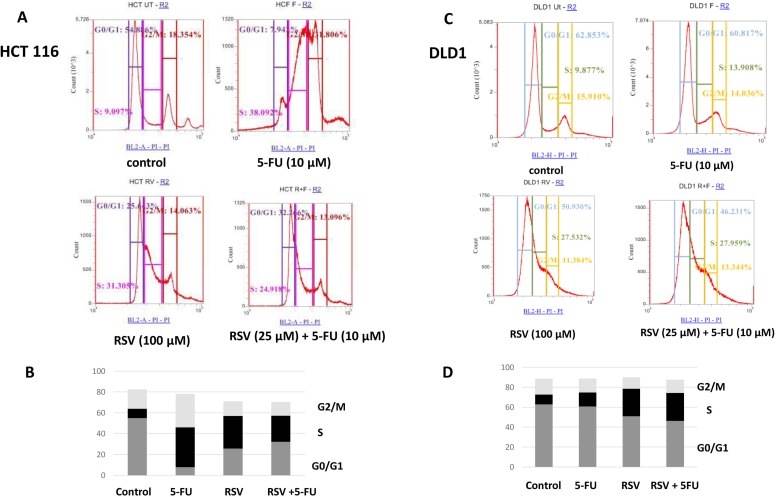
FACS analysis of cell cycle of HCT116 and DLD1 treated with 5-FU (10 μM) and resveratrol (25 μM) HCT116 and DLD1 were treated with 5-FU and resveratrol, then applied to FACS analyses for cell cycle profiling. **(A)** Representative images of cell cycle distribution of cancer cell line HCT116 with different treatments. **(B)** The distribution of cell cycle of HCT116 with different treatments **(C)** Representative images of cell cycle profiles of DLD1 with different treatment groups **(D)** The distribution of cell cycle of DLD1 with different treatments.

Next, we wished to study the cell cycle profile changes upon resveratrol and 5-FU treatments. We treated HCT116 and DLD1 cells with resveratrol and 5-FU alone and in combination for 72 hours. The cells were then stained with hypotonic staining buffer and applied to flow cytometry. In HCT116 control, G0/G1 phase was 54.8%, S phase was 9.09% and G2/M phase was 18.35% (Figure 2A). Upon 5-FU treatments, G0/G1 phase has decreased to 7.94% whereas S phase has increased to 38.09%. Similarly, resveratrol treatment has decreased G0/G1 to 25.6% while S phase has increased to 31.3%. Combined treatments with resveratrol and 5-FU also decreased G0/G1 to 32.2 % whereas S phase increased to 24.9% (Figure 2B). DLD1 showed a different cell cycle profiles with resveratrol and 5-FU treatments. DLD1 control showed G0/G1 phase of 61.3%, S phase 7.78% and G2/M phase 17.2%. 5-FU treatment alone showed the G0/G1 phase 60%, S phase 9.68% and G2/M 16%, respectively. Control and 5-FU monotherapy showed similar cell cycle profiles. However, resveratrol treatment induced S phase arrest showing increase to 44.24%. G0/G1 phase has decreased from 61.3% to 21.3% and G2/M phase slightly decreased from 17.2% to 13.8%. Combined treatments with resveratrol and 5-FU also showed S phase cell cycle arrest showing increased 41.14% (Figure 2C). G0/G1 phase has decreased to 23% and G2/M phase decreased to 15.45%. These results suggest that resveratrol alone and in combination with 5-FU resulted in S phase cell cycle arrest in human colorectal cancer cells.

### Apoptosis induction was increased by the combined resveratrol and 5-FU treatments

**Figure 3:**
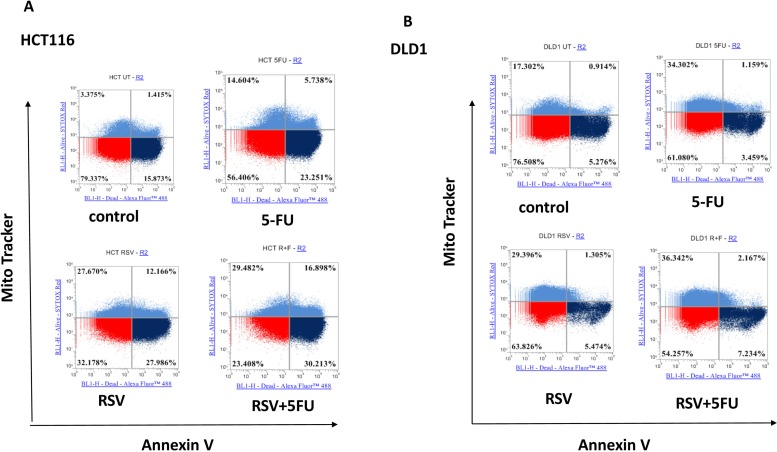
Effects of combination treatments with resveratrol and 5-FU on apoptosis of colorectal cancer cell lines Combined treatments of resveratrol (25 μM) and 5-FU (10 μM) increased apoptosis from colorectal cancer cells. **(A)** Quantification of mitochondrial depolarization and phosphatidyl serine externalization of HCT116 using mitotracker and annexin V staining. Live cells are mitotracker high/annexin V-, cells with externalized phosphatidyl serines are mitotracker high/annexin V+ and apoptotic cells are mitotracker low/annexin V+. **(B)** Quantification of mitochondrial depolarization and phosphatidyl serine externalization of DLD1.

Since S phase specific cell cycle arrest was induced, we next wished to further examine the apoptotic changes in colorectal cancer cells with resveratrol and 5-FU treatments. To this end, we treated HCT116 and DLD1 cells with resveratrol and 5-FU alone and in combination, then applied to mitochondrial potential/annexin V apoptosis assay as described in Methods and Materials. Dissipation of mitochondrial membrane potential is a sensitive marker for early apoptotic events [34]. Externalization of phosphatidyl serine is a hallmark of apoptosis [35]. In this assay, apoptotic cells show green fluorescence with decreased red fluorescence (mitotracker ^low^/annexin V+) which present a decreased mitochondrial potential and phosphatidyl serine translocation. As shown in Figure 3, HCT116 control showed 15.87% whereas 5-FU monotherapy induced 23.25 % apoptosis (Figure 3A). Furthermore, resveratrol alone induced 27.99 % and combined resveratrol and 5-FU showed 30.21 % apoptosis. These results indicate that resveratrol enhanced 5-FU's potential to induce apoptosis. When we treated DLD1 cell line, control DLD1 showed 5.28% and 5-FU treated cells showed 3.46% (Figure 3B). Apoptosis of DLD1 was practically the same with control and resveratrol treatment (5.47%). DLD1 was more resistant to resveratrol. However, the combined treatments of resveratrol and 5-FU has modestly increased apoptosis to 7.23%. Our data suggest that, when resveratrol is combined with 5-FU, the apoptosis induction is enhanced in colorectal cancer cells. This is another evidence that resveratrol augments 5-FU anti-cancer effects with increased apoptosis with a combination regimen. Our data suggest resveratrol has the potential to enhance efficacy of 5-FU chemotherapy.

### Expression levels of slug and vimentin were downregulated by the resveratrol and 5-FU combination treatments

**Figure 4:**
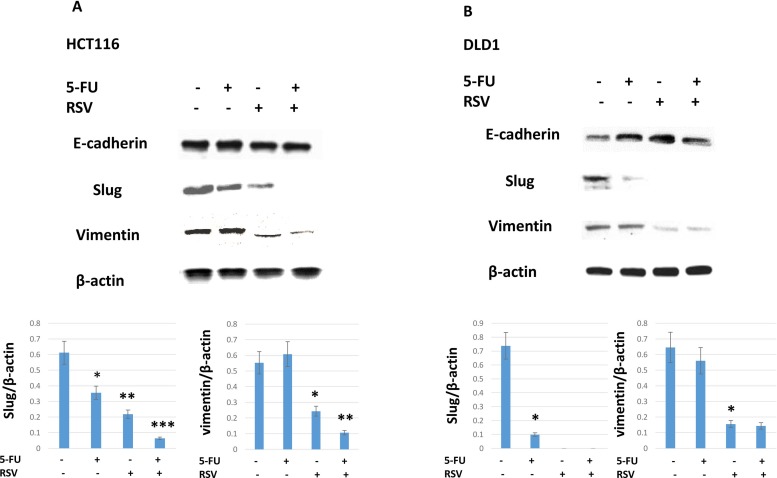
Epithelial-mesenchymal transition biomarker expressions were inhibited by the combination treatments of resveratrol and 5-FU Resveratrol and 5-FU treatment downregulated key epithelial-mesenchymal transition biomarkers of Slug and Vimentin. **(A)** HCT116 cells were treated with resveratrol and 5-FU alone and in combination. Western blot was performed to monitor the changes in slug and vimentin expression levels. Bottom panel shows slug and vimentin expression levels normalized to the β-actin control. **(B)** DLD1 cells were treated with resveratrol and 5-FU alone and in combination. Slug and vimentin were examined for expression levels normalized to β-actin control.

Next we wished to test whether combined treatments of resveratrol and 5-FU inhibit the expression of slug and vimentin. Slug is a transcription factor that plays a role in epithelial-mesenchymal transition (EMT). EMT is positively correlated with cancer invasiveness and resistance to chemotherapy. Vimentin is another well-established mesenchymal biomarker. In HCT116, both slug and vimentin were downregulated with resveratrol treatments (Figure 4A). When combined with 5-FU, resveratrol abolished the slug and vimentin expressions. Slug and vimentin expression levels were normalized by β-actin protein loading controls. On the other hand, epithelial biomarker E-cadherin expression levels remained the same with the resveratrol alone and in combination with 5-FU treatments in HCT116. In DLD1, slug regulation was more sensitive to either 5-FU or resveratrol monotherapies and in combination treatments. Resveratrol monotherapy clearly reduced both slug and vimentin expressions (Figure 4B). E-cadherin expression was modestly increased in DLD1 cell line. Our data suggest that EMT driver slug and mesenchymal marker vimentin expressions were downregulated by resveratrol and 5-FU combination treatments.

### Migration capacity was abolished by the combination treatments with resveratrol and 5-FU

**Figure 5:**
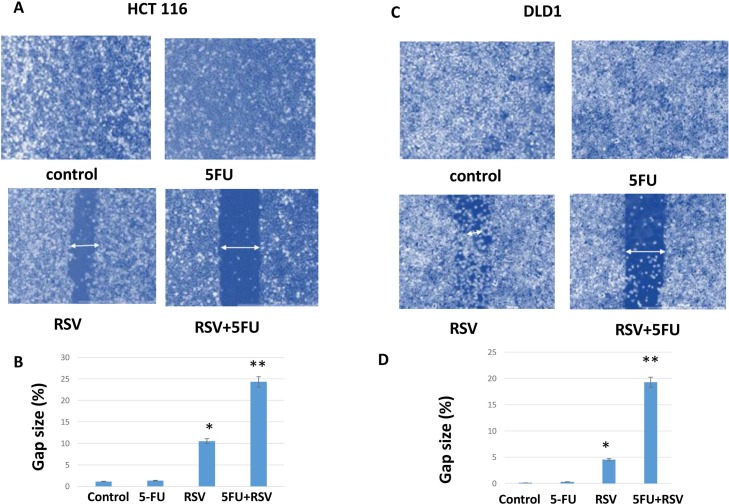
Wound healing assay of HCT116 and DLD1 treated with resveratrol and 5-FU Combination treatments with resveratrol and 5-FU reduced the wound healing capability of colorectal cancer cells. **(A)** HCT116 untreated control, pre-treated with 5-FU for 24 hours, pre-treated with resveratrol and pre-treated with combined treatments of resveratrol and 5-FU were examined for healing capacity after wound induction as a migration capacity. **(B)** DLD1 untreated control, pre-treated with 5-FU, resveratrol alone and in combination cells were monitored for wound healing capacity after the wound induction.

To measure the cell migration ability, we employed wound healing assay. HCT116 and DLD1 cancer cells were pre-treated for 24 hours with resveratrol and 5-FU alone and in combination. Cells were then plated and incubated to a full confluency. A lawn of cells were scratched with a fine tip, and wound healing closure size was measured for areas between two layers of scratches. HCT116 cells scratch assay revealed that resveratrol treatment inhibited the wound healing leaving the gap size of 10.5% and combined resveratrol and 5-FU treatments even further inhibited the healing resulting in the gap area of 24.3% (Figure 5A and 5B). Control and 5-FU monotherapy did not inhibit the cell migration in HCT116 cells. Similarly, in DLD1 cells, combined treatments inhibited the wound healing to the gap size of 19.3% whereas resveratrol alone showed 4.5% gap size (Figure 5C and 5D). Control and 5-FU monotherapy did not inhibit the migration ability of DLD1 cells. These results suggest that combined resveratrol and 5-FU enhanced the inhibitory effects on the migration of human colorectal cancer cells.

### Combined treatments with resveratrol and 5-FU decreased the cancer stem-like cell phenotype

**Figure 6:**
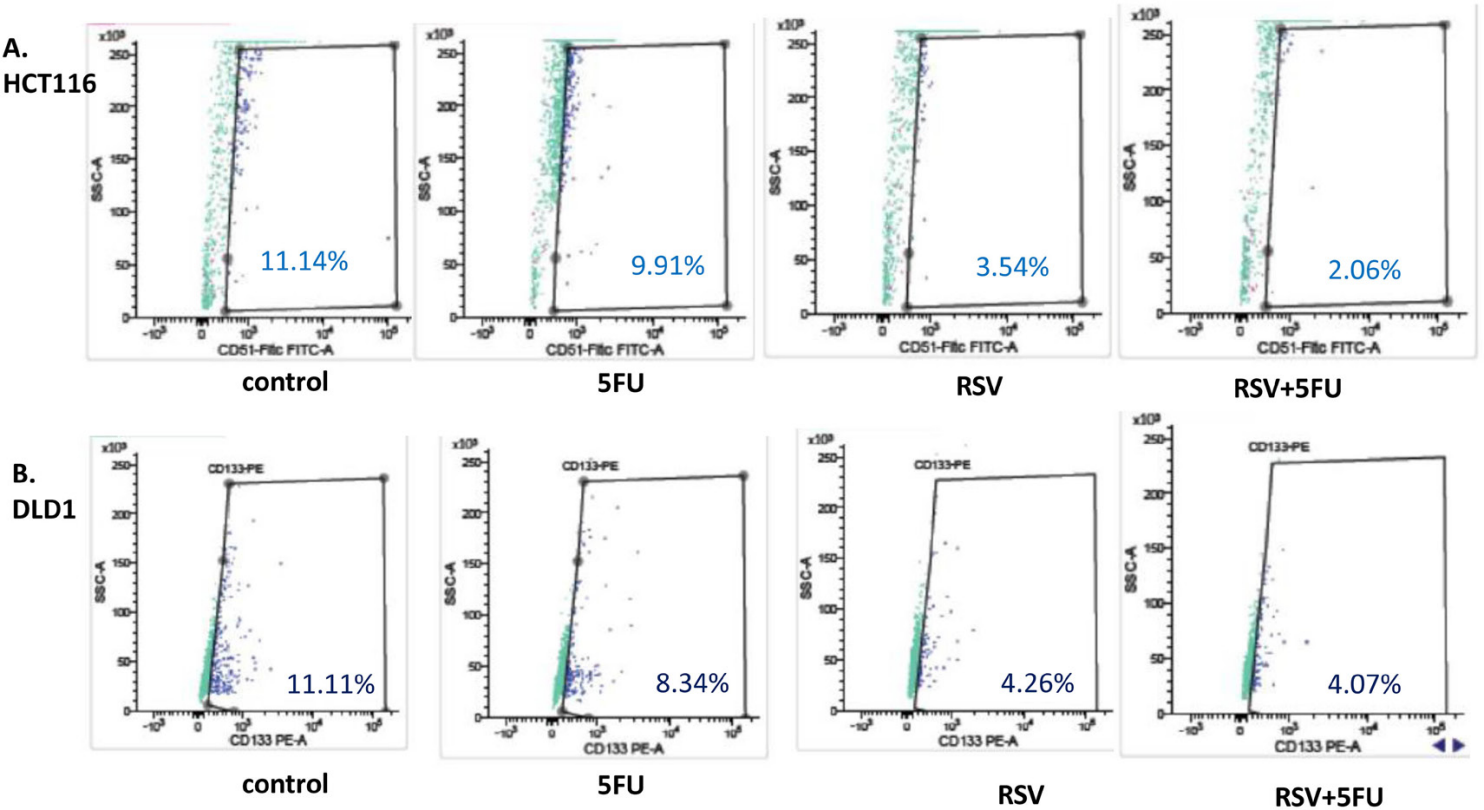
Cancer stem cell biomarker CD51 was decreased with resveratrol and 5-FU combination treatments To determine the anti-cancer stemness effects of resveratrol and 5-FU combined treatments, HCT116 and DLD1 cells were treated with resveratrol and 5-FU alone and in combination for 48 hours, then applied to flow cytometry analysis for CD51-FITC. **(A)** HCT116 cells were treated with 5-FU and resveratrol alone and in combination, then applied to flow cytometry analyses. **(B)**. DLD1 cells were treated with 5-FU and resveratrol alone and in combination, then applied to flow cytometry analyses.

Next, we wished to test whether resveratrol and 5-FU treatments inhibited cancer stem cell-like phenotype in colon cancer cells. For HCT116, CD51 is a novel biomarker for colon cancer stemness [36]. We treated HCT116 cells with resveratrol alone and in combination with 5-FU, stained with CD51-Fitc antibody and applied to the flow cytometric analyses. As shown in the figure, control HCT116 showed 11.14% positivity of CD51 (Figure 6A). When treated with 5-FU, CD51 positivity was 9.91% and resveratrol treatments reduced the CD51 to 3.54%. When combined with 5-FU, resveratrol showed 2.06% CD51 positivity. In DLD1, resveratrol treatments reduced the CD51 (+) populations from 11.11% to 4.26% (Figure 6B) and when combined with 5-FU, the CD51 (+) populations reduced to 4.07%. Our data suggest that resveratrol can inhibit the cancer stemness and the inhibitory effects can be enhanced with 5-FU combinations.

### Resveratrol showed anti-inflammatory effects with time-dependent inhibition of pSTAT3 and pNFkB

**Figure 7:**
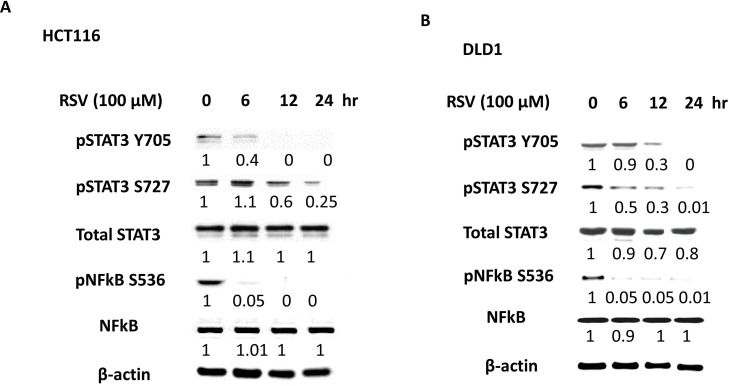
Anti-inflammatory effects of resveratrol with pSTAT3 and pNFkB inhibition To verify the anti-inflammatory effects of resveratrol, HCT116 and DLD1 were treated with resveratrol (100 μM) over the time course of 0, 6, 12 and 24 hours. **(A)** In HCT116, inflammation biomarkers pSTAT3 Y705, pSTAT3 S727 and pNFkB S were monitored over the resveratrol treatment on a time course. **(B)** DLD1 was treated with resveratrol (100 μM) over 24 hours and examined for inflammation biomarker status. Next, colorectal cancer cells were treated with either 5-FU or resveratrol alone or combined, then the key proteins were analyzed by western blotting. Densiometric values of protein bands were corrected based on β-actin and were expresses relative to that of untreated control cell, which was set as 1.0.

Inflammation is known to drive cancer progression and promote metastasis especially in colorectal cancer. We tested whether resveratrol can reduce the inflammation biomarkers in our system. We used pSTAT3 (Y705 and S727) and pNFkB (S536) as inflammation monitoring markers. In HCT116 cells, pSTAT3 (Y705) and pSTAT3 (S727) were inhibited at the time point of 12 hours (Figure 7A) and pNFkB (S536) was inhibited at the point of 6 hours. In DLD1, both pSTAT3, Y705 and S727 were inhibited at the time point of 12 hours and pNFkB was inhibited at the time point of 4 hours (Figure 7B). Our data suggest that resveratrol treatments show the anti-inflammatory effects on a time-dependent manner. Next, we wished to study the STAT3 and Akt phosphorylation status with the resveratrol alone and in combination with 5-FU.

### Combined treatments of resveratrol and 5-FU concurrently abolished CD44, STAT3 and Akt activation

**Figure 8:**
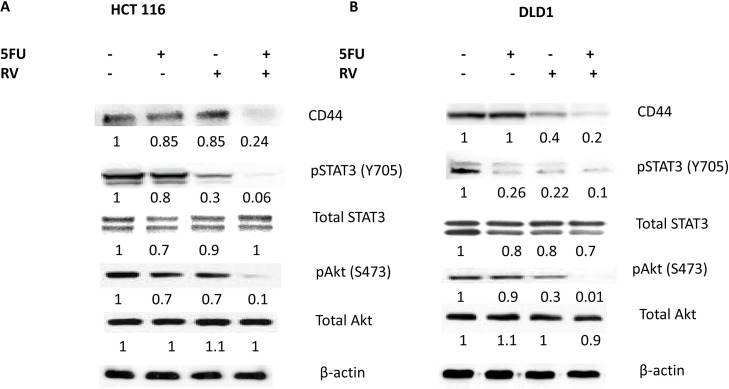
Combined treatments of resveratrol and 5-FU abolished the CD44, pSTAT3 and pAkt signaling Cancer stem cell biomarker CD44, STAT3 and Akt expressions were examined for their expression levels. **(A)** HCT116 untreated control and treated with 5-FU alone (10 μM), treated with resveratrol alone (25 μM) and treated with 5-FU and resveratrol in combination were applied to the western analyses of CD44, pSTAT3, total STAT3, pAkt and total Akt. **(B)** DLD1 untreated control, treated with 5-FU alone (10 μM), treated with resveratrol alone (25 μM) and treated with 5-FU and resveratrol combination were applied to the western analyses. Densiometric values of western bands were normalized to a β-actin loading control, then calculated relative to the value of untreated cells, which was set as 1.0.

CD44 is well established cancer stem cell biomarker and expressed in both HCT116 and DLD1 cell lines [37]. To test whether combination treatments of resveratrol and 5-FU abolish CD44 expressions, we treated the colorectal cancer cell lines with mono- and combo-therapies of resveratrol and 5-FU, then performed western blot analysis. As shown in Figure 8, neither resveratrol nor 5-FU monotherapy abolished CD44 expression. However, when we treated cancer cells with the combination, CD44 expression was abolished (Figure 8A and 8B). STAT3 is involved in immune response and aberrantly expressed in colorectal cancer cells. We treated the cancer cells with resveratrol and 5-FU and examined the STAT3 phosphorylation status at Tyrosine 705. Activated STAT3 (pSTAT3) was inhibited by the combination treatments of resveratrol and 5-FU both in HCT116 and DLD1 cells. Total STAT3 levels mainly remain unchanged. Akt is a well-known serine/threonine kinase that plays a key role in cell proliferation, survival and migration [38]. Since resveratrol and 5-FU combination inhibited cell proliferation and migration, we wished to determine the Akt phosphorylation status upon these treatments. Phosphorylated Akt was inhibited with the combined treatments of resveratrol and 5-FU. Total Akt expression levels were not changed. Our data suggest resveratrol and 5-FU combination efficiently abolish CD44 expression and inhibit the key kinase Akt and transcription factor STAT3.

### Resveratrol enhanced anti-telomeric activities of 5-FU by inhibiting STAT3 binding to hTERT promoter region

**Figure 9:**
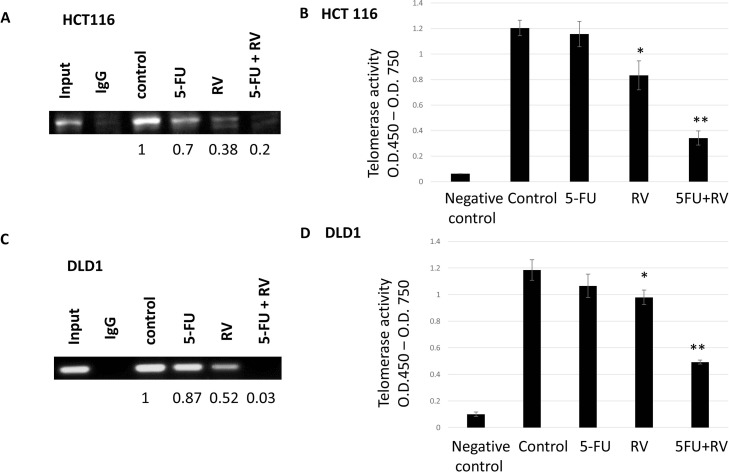
STAT3 binding to promoter region hTERT were inhibited by the resveratrol and 5-FU alone and in combination and corresponding telomerase activities were decreased upon the combination treatments Transcription factor STAT3 binding to hTERT promoter region was tested with chromatin immunoprecipitation assay. **(A)** HCT116 was treated with 5-FU and resveratrol alone and in combination and applied to ChIP assay. STAT3 binding was quantified by the ChIP band intensity relative to untreated control, which was set as 1.0. **(B)** Telomerase activities were measured by TRAP PCR reaction conjugated to Elisa assay. **(C)** DLD1 was treated with 5-FU (10 μM) alone and associated with 25 μM resveratrol combination, then applied to ChIP assay to quantify the STAT3 binding. **(D)** DLD1 was treated with 5-FU and resveratrol alone and in combination and applied to TRAP-PCR-Elisa assay to quantify telomerase activities. Error bars represent standard deviation. Telomerase activities were measured three times independently.

We have previously shown that STAT3 was transcriptionally regulating telomerase reverse transcriptase (hTERT) in human breast cancer cells [39]. In this study, we wished to determine whether transcription factor STAT3 bound to hTERT promoter region in colorectal cancer cell lines. We also tested whether resveratrol alone and in combination with 5-FU can inhibit STAT3 binding to the hTERT promoter region. To this end, we have performed the chromatin immunoprecipitation (CHIP) assay as described in the Materials and Methods section. Consensus STAT3 binding sites (TTCNNNGAA) reside within the hTERT promoter. Chip assay was performed on the first STAT3-binding sites. Cells were treated with 5-FU and resveratrol alone and combined. In HCT116, we found STAT3 binding affinity was reduced to 0.7 fold compared to the untreated control upon 5-FU treatments (Figure 9A). Resveratrol treatments also decreased the STAT3 binding to 0.38 folds. When we treated the HCT116 with resveratrol and 5-FU in combination, STAT3 binding was reduced to 0.2 folds compared to untreated control. Since STAT3 is regulating hTERT transcriptionally, we next examined the telomerase activities with the combination treatments. HCT116 was treated with resveratrol and 5-FU alone and in combination, then applied to telomerase activity assay where telomere DNA PCR was performed in conjugated with Elisa assay. 5-FU alone did not change the telomerase whereas resveratrol has decreased telomerase from 1.2 of control to 0.81, and when combined with 5-FU, telomerase activity was decreased to 0.37 (Figure 9B). It is approximately 70% telomerase activity reduction. Similarly, in DLD1, STAT3 binding to hTERT promoter was decreased to 0.87 and 0.52 when treated with 5-FU and resveratrol alone, respectively. However, the combination treatments reduced the STAT3 binding to 0.03 comparing with untreated control (Figure 9C). In DLD1, telomerase has decreased from 1.19 of control to 0.95 with resveratrol treatments (Figure 9D). Furthermore, resveratrol and 5-FU treatments has reduced the telomerase from 1.19 to 0.47 and it is about 61% activity reduction. Combination treatments with resveratrol and 5-FU inhibited STAT3 binding, and in accordance, telomerase activity has been reduced.

**Figure 10:**
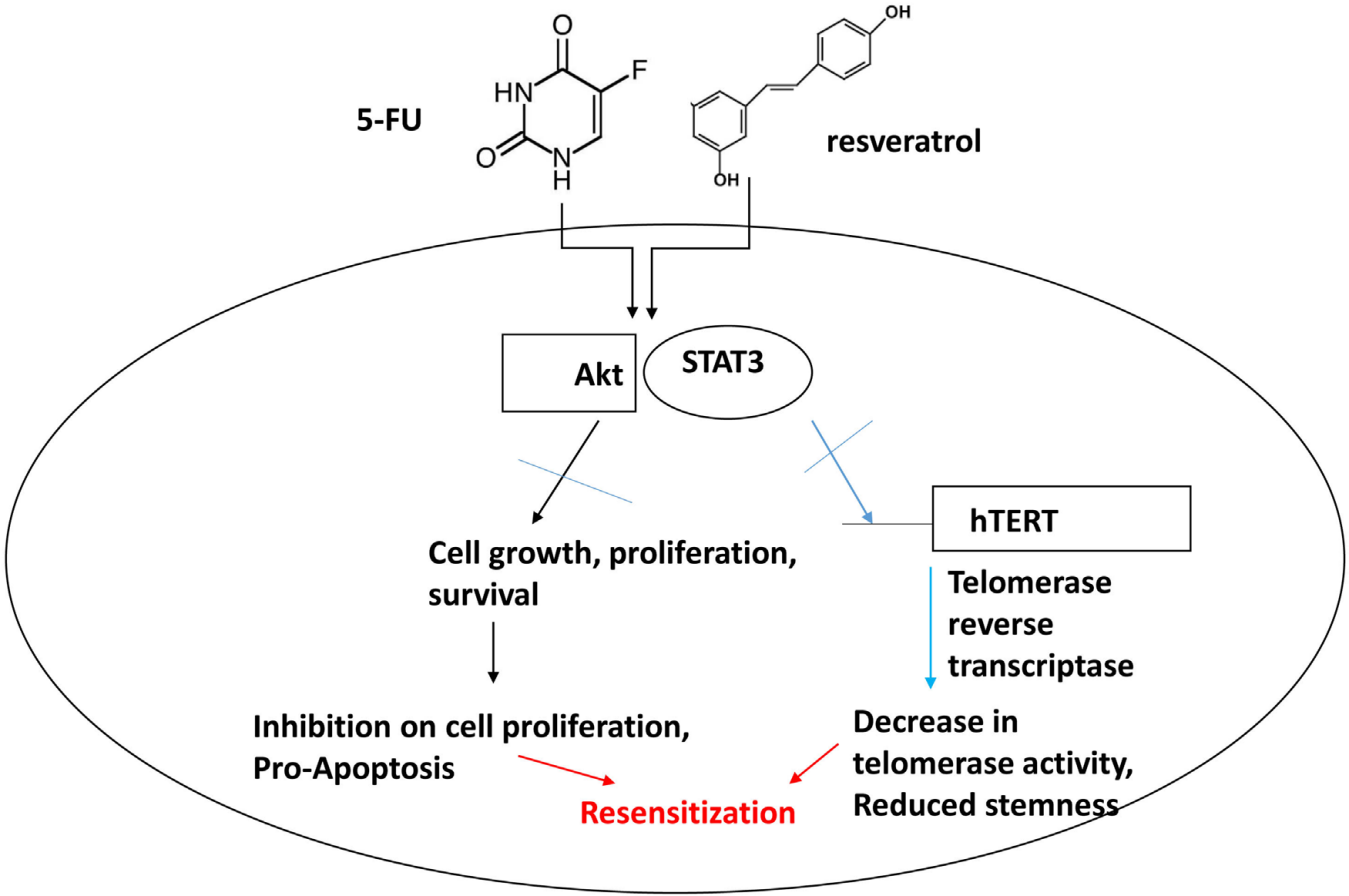
Schematic representation of combination treatment effects with 5-FU and resveratrol in colorectal cancer cells The combined treatments with 5-FU and resveratrol inhibit Akt and STAT3 signaling pathways in colorectal cancer. Akt plays a key role in cell proliferation and survival and STAT3 is key transcription factor for telomerase and other target genes involved in immune response and potential stem-like traits. Our model suggests that combined treatments of 5-FU and resveratrol drive apoptosis by inhibiting Akt and STAT3, concurrently decreasing telomerase activity and downregulating target genes of STAT3 leading to re-sensitization of colorectal cancer to chemotherapy.

Our study showed that combination treatments of resveratrol and 5-FU inhibited STAT3 and Akt phosphorylation in human colorectal cancer cells. Deactivated STAT3 resulted in decreased binding to the hTERT promoter, subsequently reduced telomerase activity which is important to maintain chromosomal stability over repeated cell division. Combined resveratrol and 5-FU also inhibited Akt phosphorylation which caused the anti-proliferative and pro-apoptotic progression in colorectal cancer. Schematic model for our study has been presented (Figure 10). In summary, our data suggest that chemotherapy agent combined with natural compound resveratrol can be a novel targeted-therapeutic modalities for advanced colorectal cancer.

## DISCUSSION

Resveratrol has been shown to have many beneficial biological properties including cancer chemopreventive and therapeutic effects [40]. We tested human colorectal cancer cell lines HCT116 and DLD1 for resveratrol IC50 and found the differences between them. HCT116 has a RAS mutation whereas DLD1 has a p53 missense mutation (Ser241 → Phe) [32]. The p53 mutation in DLD1 may cause the high expression of p53 and contribute to the higher IC50 of resveratrol. It has been reported that resveratrol exerts differential effects on proliferation of cancer cells from different origin which is mainly accompanied by p53 activation [33].

In current study, we cultured colon cancer cell lines in DMEM/F12 with 10% FBS (fetal bovine serum) and examined resveratrol's anti-proliferative effects. Since bovine-source serum contained thyroid hormone, there was a possibility that FBS reduced the apoptotic potential of resveratrol. However, without growth factors' stimulation, cancer cells may undergo apoptosis, hence we used the culture media with FBS.

In spite of its beneficial biological functions, resveratrol is a natural compound and has certain limitations in curing multiple diseases as a monotherapy, especially for cancer. 5-Fluorouracil has been widely used for various solid tumors including colorectal cancer over 60 years functioning as an antimetabolite. To date, adjuvant chemotherapy remains the first-line therapy for colorectal cancer treatments [41]. However, frequent drug resistance, toxicity and disease recurrence hampered 5-FU's extensive applications in clinical setting [42]. Numerous *in vitro* and *in vivo* studies have suggested that resveratrol may enhance the antitumor activity of chemotherapy in several cancers [43]. Specifically, in colorectal cancer, chronic inflammation promotes the initiation and progression of colon cancer [44], hence resveratrol's anti-inflammatory effects may function synergistically with other drugs. We tested herein whether resveratrol might enhance cytotoxicity of 5-FU for colorectal cancer and further potentiate its anti-telomeric activities. Since resveratrol showed clear anti-inflammatory effects, we wished to determine if it can work with 5-FU in a potency-enhancing manner. In addition, resveratrol is known to interact with numerous target molecules through the simple chemical structure [45]. Combined treatments with resveratrol and 5-FU enhanced anti-proliferative effects, induced the S phase cell cycle arrest and increased apoptosis. Moreover, the combined treatments inhibited pAkt and pSTAT3 signaling and concurrently reduced telomerase activity. Akt is known to play roles in cell proliferation, survival and drug resistance [46]. The cell cycle arrest and increased apoptosis may have been caused by the cumulative effects of resveratrol and 5-FU impact on the cancer cells. Our data suggest that resveratrol combined chemotherapy regimen can be a novel efficient therapeutic modalities for advanced colorectal cancer.

Telomerase is hyper-activated in over 90% of human malignancies including colorectal cancer. We have shown the first evidence of resveratrol potentiating anti-telomeric activities of 5-FU in colorectal cancer. Telomerase is also known to have a non-canonical function which is driving stemness in the cancer cells, playing a role as a transcription co-factor in transcriptional regulation of Wnt signaling pathway [47]. We have previously shown that STAT3 is regulating telomerase expressions in aggressive cancer cells [39]. We report here the combined treatments of resveratrol and 5-FU inhibits STAT3 activation and deceased telomerase activity. This is consistent that cancer stem cell marker CD44 is abolished upon the combination treatments since telomerase drives stemness in cancer and stem cells. We will pursue the STAT3-telomerase-cancer stem cell axis in colorectal cancer in the next phase. More *in vivo* studies are under way to confirm this STAT3-telomerase axis and its role in cancer stem cells.

Resveratrol has been shown to enhance various chemotherapeutic drugs in several cancers. It has been reported that resveratrol potentiated an alkylating agent temozolomide in a mouse xenograft model of glioma [48]. In there, combination treatments inhibited ROS/MEK mediated autophagy and enhanced apoptosis. It has been also reported that resveratrol overcame chemoresistance in a mouse model of B16/DOX melanoma and prolonged survival of mice [49]. Finally, a combination of resveratrol, quercetin and catechin potentiated the gefitinib effects in nude mice to inhibit the mammary tumor growth and metastasis [50]. However, there is a contradictory report that resveratrol diminished the anti-proliferative effect of paclitaxel in mice [51]. These studies suggest that there might be either synergistic, additive or antagonistic interactions between resveratrol and different chemotherapeutic agents. In case of antagonistic interactions, one possibility is resveratrol might have interfered or competed with the counterpart chemotherapeutic agent in cell absorption. It remains to be seen more preclinical studies in proper models to ascertain the clinical importance of resveratrol when combining with various conventional chemotherapeutic agents. Our study present more evidence that resveratrol can be used as an adjuvant for chemotherapeutic agent 5-FU with an enhanced efficacy. We expect to see the reduced toxicity and resistance by adding resveratrol to the chemotherapy in clinical trials due to its augmenting effects on 5-FU. Our data also suggest that resveratrol combined 5-FU enhance anti-proliferative effects with pAkt inhibition and can reduce the telomerase activity with pSTAT3 inhibition. Taken altogether, resveratrol combination treatments can be a novel, enhanced targeted-therapy for human colorectal cancer.

## MATERIALS AND METHODS

### Cell lines and reagents

DLD1 and HCT116 colon cancer cell lines were purchased from the American Type Culture Collection (ATCC, Manasas, VA, U.S.A.). HCT116 and DLD1 cells were maintained in a monolayer culture in DMEM/F12 (Dulbecco's modified Eagle medium) with 10% fetal bovine serum, 2.5% L-Glutamine and 0.5% Penicillin/Streptomycin. 5-Flurouracil (Sigma, catalog number: F6627) and resveratrol (Sigma, catalog number: R5010) were purchased from Sigma company (St. Louis, MO, USA). 5-FU stock solution was made at the 10 mM in distilled water and resveratrol stock solution was made at the concentration of 100 mM in dimethyl sulfoxide (DMSO), respectively.

### Cell proliferation MTS assay

Cancer cells were cultured in 96-well plates (2 × 10^4^ cells per well) at 37°C in a 5 % CO_2_ incubator. Cells were then treated with various concentrations of resveratrol alone or in combination with 5-FU (10 μM). The cell viability was determined using the 96 Aqueous One Solution Cell Proliferation Assay kit [MTS, 3-(4,5-dimethylthiazol-2-yl)-5-(3-car boxymethoxyphenyl)-2-(4-sulfophenyl)-2H-tetrazolium] (Promega, catalog number: G3580, Madison, WI, USA) following the manufacturer's instructions. The quantity of formazan product was determined by measuring absorbance at 450 nm using a Promega Glo Max-Multi detection system (Promega, Madison, WI).

### Cell cycle analysis

Cell cycle profiles were analyzed using a flow cytometric DNA method described previously [52]. Briefly, cells were treated with 5-FU and/or resveratrol for 72 hours. Cells were harvested, then incubated with 0.5 ml of hypotonic staining buffer (sodium citrate 0.25 g, Triton-X 100 0.75 ml, propium iodide 0.025 g, ribonuclease A 0.005 g and distilled water 250 ml) for 15 minutes or for a maximum of 1 hour before acquisition on the flow cytometer. The cells were acquired and analyzed with Attune NxT flow cytometer (Thermo Fisher Scientific, U.S.A.).

### Analysis of apoptosis

Mitochondrial potential and phosphatidyl serine externalization were evaluated using Mitochondrial Membrane Potential Apoptosis kit with Mitotracker TM Red and Annexin V Alexa Fluor 488 (Thermo Fisher Catalog number: V35116) following the manufacturer's instructions. The cells were treated with resveratrol and 5-FU alone and in combination for 72 hours and processed with the Apoptosis kit and analyzed with the Attune NxT flow cytometer.

### Western blot analysis

Monolayer cultures of respective cell lines at 80-90% confluence were lysed using 100 μl of RIPA buffer (Thomas Scientific Inc. Swedesboro, NJ). Tris-glycine (Bio-Rad, Irvine, CA) pre-cast gels were loaded with 50-100 μg of cell lysates. After electrophoresis, the gel was transferred to a nitrocellulose membrane for 1 hour. The membrane was blocked for 1 hour in 5% skim milk at 4°C. The membrane was then washed 3 times with 1x TTBS and incubated overnight with the primary antibody at 4°C. Primary antibodies of STAT3, pSTAT3, pAkt, Akt, CD44 and β-actin were purchased from Cell Signaling Technology (Danvers, MA). After incubation with the secondary antibodies conjugated with horseradish peroxidase (HRP), the protein bands were developed with the chemiluminescent reagents.

### Chromatin immunoprecipitation assay

Chromatin immunoprecipitation (ChIP) Assay Kit (Millipore, Catalog number: 17-295) was utilized to study STAT3 binding to hTERT promoter region. DLD-1 or HCT116 cells were incubated with 1% formaldehyde for 20 minutes at 37°C. Cells were collected, lysed, sonicated, and incubated with 4 μg of antibodies to STAT3 overnight. PCR was used to amplify DNA bound to the immunoprecipitated histones after reversing the histone-DNA cross-links. Primer sets were designed flanking the possible STAT3 binding regions. Primer sequences: *hTERT* promoter primer sequence 1, forward primer 5′-CCAAACCTGTGGACAGAACC-3′ and reverse primer 5′-AGACTGACTGCCTCCATCGT-3′ and *hTERT* promoter primer sequence 2, forward primer 5′-GGGGTGTCTTCTGGGTATCA-3′ and reverse primer 5′-AAGGGCTGTGTTTGTGAATTG-3′. PCR products of Chip assays were resolved on a 2.5% agarose gels. The STAT3 bound band densities were quantified by using soft ware Image J.

### Telomerase activity assays

Cancer cells were processed according to the manufacturer's protocol for the TeloTAGGG Telomerase PCR ELISA kit (Roche, Orange, CA. Catalog number: 11854666910). Briefly, cell pellets were thawed in lysis reagent, incubated on ice for 30 minutes, and centrifuged at 16,000 *g* for 20 minutes at 4°C. Telomerase activity was immediately measured in the resultant supernatant using the telomeric repeat amplification protocol in which telomerase, if present in the cell lysate, adds telomeric repeats to the 3′ end of a biotin-labeled synthetic P1-TS primer. Samples were amplified by polymerase chain reaction (PCR), with P1-TS and P2 primers creating an elongated telomere. The PCR product was denatured and hybridized to a digoxigenin-labeled probe that detects telomeric repeats in a subsequent enzyme-linked immunosorbent assay (ELISA). Telomerase assays were performed three times independently and P values less than 0.05 were considered statistically significant.

### Statistical analysis

Student t-tests were used to evaluate the significance of changes in all combination treatment assays compared to controls. Data collected from each experiment was used to calculate the mean values and standard deviations (SD). Experiments were repeated three times independently. Differences were considered statistically significant if *P* < 0.05.

## Abbreviations

RSV: Resveratrol
5-FU: 5 Fluorouracil
STAT3: Signal transducer and activator of transcription 3
NFκB: Nuclear factor κB
hTERT: human telomerase reverse transcriptase
CD44: Cluster of differentiation 44

## References

[1] Siegel R, Ma J, Zou Z, Jemal A (2014). Cancer statistics, 2014. CA Cancer J Clin.

[2] Helling TS, Martin M (2014). Cause of death from liver metastases in colorectal cancer. Ann Surg Oncol.

[3] Carrie L, Arrington Amanda K, Hans FS, Gagandeep S, Joseph K (2013). Targeted therapies in colorectal cancer: surgical considerations. Gastrointest Oncol.

[4] Mishra J, Drummond J, Quazi SH, Karanki SS, Shaw JJ, Chen B, Kumar N (2013). Prospective of colon cancer treatments and scope for combinatorial approach to enhanced cancer cell apoptosis. Crit Rev Oncol Hematol.

[5] Kurkjian C, Kummar S (2009). Advances in the treatment of metastatic colorectal cancer. Am J Ther.

[6] Papamichael D (1999). The use of thymidylate synthase inhibitors in the treatment of advanced colorectal cancer: current status. Oncologist.

[7] Liu HC, Chen GG, Vlantis AC, Leung BC, Tong MC, van Hasselt CA (2006). 5-fluorouracil mediates apoptosis and G1/S arrest in laryngeal squamous cell carcinoma via a p53-independent pathway. Cancer J.

[8] Bastos DA, Ribeiro SC, de Freitas D, Hoff PM (2010). Combination therapy in high-risk stage II or stage III colon cancer: current practice and future prospects. Ther Adv Med Oncol.

[9] Panczyk M (2014). Pharmacogenetics research on chemotherapy resistance in colorectal cancer over the last 20 years. World J Gastroenterol.

[10] Varghese A (2015). Chemotherapy for stage II colon cancer. Clin Colon Rectal Surg.

[11] Pandey KB, Rizvi SI (2009). Plant polyphenols as dietary antioxidants in human health and disease. Oxid Med Cell Longev.

[12] Habauzit V, Morand C (2012). Evidence for a protective effect of polyphenols-containing foods on cardiovascular health: an update for clinicians. Ther Adv Chronic Dis.

[13] Kulkarni SS, Cantó C (2015). The molecular targets of resveratrol. Biochim Biophys Acta.

[14] Carter LG, D'Orazio JA, Pearson KJ (2014). Resveratrol and cancer: focus on in vivo evidence. Endocr Relat Cancer.

[15] Nowsheen S, Yang ES (2012). The intersection between DNA damage response and cell death pathways. Exp Oncol.

[16] Lin HY, Tang HY, Keating T, Wu YH, Shih A, Hammond D, Sun M, Hercbergs A, Davis FB, Davis PJ (2008). Resveratrol is pro-apoptotic and thyroid hormone is anti-apoptotic in glioma cells: both actions are integrin and ERK mediated. Carcinogenesis.

[17] Lin HY, Lansing L, Merillon JM, Davis FB, Tang HY, Shih A, Vitrac X, Krisa S, Keating T, Cao HJ, Bergh J, Quackenbush S, Davis PJ (2006). Integrin alphaVbeta3 contains a receptor site for resveratrol. FASEB J.

[18] Alayev A, Salamon RS, Schwartz NS, Berman AY, Wiener SL, Holz MK (2017). Combination of rapamycin and resveratrol for treatment of bladder cancer. J Cell Physiol.

[19] Cai H, Scott E, Kholghi A, Andreadi C, Rufini A, Karmokar A, Britton RG, Horner-Glister E, Greaves P, Jawad D, James M, Howells L, Ognibene T (2015). Cancer chemoprevention: evidence of a nonlinear dose response for the protective effects of resveratrol in humans and mice. Sci Transl Med.

[20] Zhong Z, Wen Z, Darnell JE (1994). Stat3: a STAT family member activated by tyrosine phosphorylation in response to epidermal growth factor and interleukin-6. Science.

[21] Carpenter RL, Lo HW (2014). STAT3 target genes relevant to human cancers. Cancers (Basel).

[22] Hirano T, Ishihara K, Hibi M (2000). Roles of STAT3 in mediating the cell growth, differentiation and survival signals relayed through the IL-6 family of cytokine receptors. Oncogene.

[23] Gilmore TD (2006). Introduction to NF-kappaB: players, pathways, perspectives. Oncogene.

[24] Lawrence T (2009). The nuclear factor NF-kappaB pathway in inflammation. Cold Spring Harb Perspect Biol.

[25] Banerjee K, Resat H (2016). Constitutive activation of STAT3 in breast cancer cells: A review. Int J Cancer.

[26] Bertorelle R, Rampazzo E, Pucciarelli S, Nitti D, De Rossi A (2014). Telomeres, telomerase and colorectal cancer. World J Gastroenterol.

[27] Tatsumoto N, Hiyama E, Murakami Y, Imamura Y, Shay JW, Matsuura Y, Yokoyama T (2000). High telomerase activity is an independent prognostic indicator of poor outcome in colorectal cancer. Clin Cancer Res.

[28] Jafri MA, Ansari SA, Alqahtani MH, Shay JW (2016). Roles of telomeres and telomerase in cancer, and advances in telomerase-targeted therapies. Genome Med.

[29] Cong YS, Wright WE, Shay JW (2002). Human telomerase and its regulation. Microbiol Mol Biol Rev.

[30] Liu Z, Li Q, Li K, Chen L, Li W, Hou M, Liu T, Yang J, Lindvall C, Björkholm M, Jia J, Xu D (2013). Telomerase reverse transcriptase promotes epithelial-mesenchymal transition and stem cell-like traits in cancer cells. Oncogene.

[31] Maida Y, Masutomi K (2015). Telomerase reverse transcriptase moonlights: therapeutic targets beyond telomerase. Cancer Sci.

[32] Rodrigues NR, Rowan A, Smith ME, Kerr IB, Bodmer WF, Gannon JV, Lane DP (1990). p53 mutations in colorectal cancer. Proc Natl Acad Sci USA.

[33] Amiri F, Zarnani AH, Zand H, Koohdani F, Jeddi-Tehrani M, Vafa M (2013). Synergistic anti-proliferative effect of resveratrol and etoposide on human hepatocellular and colon cancer cell lines. Eur J Pharmacol.

[34] Wlodkowic D, Skommer J, Darzynkiewicz Z (2009). Flow cytometry-based apoptosis detection. Methods Mol Biol.

[35] Balasubramanian K, Mirnikjoo B, Schroit AJ (2007). Regulated externalization of phosphatidylserine at the cell surface: implications for apoptosis. J Biol Chem.

[36] Wang J, Zhang B, Wu H, Cai J, Sui X, Wang Y, Li H, Qiu Y, Wang T, Chen Z, Zhu Q, Xia H, Song W, Xiang AP (2017). CD51 correlates with the TGF-beta pathway and is a functional marker for colorectal cancer stem cells. Oncogene.

[37] Sahlberg SH, Spiegelberg D, Glimelius B, Stenerlöw B, Nestor M (2014). Evaluation of cancer stem cell markers CD133, CD44, CD24: association with AKT isoforms and radiation resistance in colon cancer cells. PLoS One.

[38] Liu P, Cheng H, Roberts TM, Zhao JJ (2009). Targeting the phosphoinositide 3-kinase pathway in cancer. Nat Rev Drug Discov.

[39] Chung SS, Aroh C, Vadgama JV (2013). Constitutive activation of STAT3 signaling regulates hTERT and promotes stem cell-like traits in human breast cancer cells. PLoS One.

[40] Singh CK, Ndiaye MA, Ahmad N (2015). Resveratrol and cancer: challenges for clinical translation. Biochim Biophys Acta.

[41] Wolpin BM, Mayer RJ (2008). Systemic treatment of colorectal cancer. Gastroenterology.

[42] Kuczynski EA, Sargent DJ, Grothey A, Kerbel RS (2013). Drug rechallenge and treatment beyond progression—implications for drug resistance. Nat Rev Clin Oncol.

[43] Singh CK, George J, Ahmad N (2013). Resveratrol-based combinatorial strategies for cancer management. Ann N Y Acad Sci.

[44] Grivennikov SI (2013). Inflammation and colorectal cancer: colitis-associated neoplasia. Semin Immunopathol.

[45] Tsai HY, Ho CT, Chen YK (2017). Biological actions and molecular effects of resveratrol, pterostilbene, and 3′-hydroxypterostilbene. Yao Wu Shi Pin Fen Xi.

[46] Fresno Vara JA, Casado E, de Castro J, Cejas P, Belda-Iniesta C, González-Barón M (2004). PI3K/Akt signalling pathway and cancer. Cancer Treat Rev.

[47] Park JI, Venteicher AS, Hong JY, Choi J, Jun S, Shkreli M, Chang W, Meng Z, Cheung P, Ji H, McLaughlin M, Veenstra TD, Nusse R (2009). Telomerase modulates Wnt signalling by association with target gene chromatin. Nature.

[48] Lin CJ, Lee CC, Shih YL, Lin TY, Wang SH, Lin YF, Shih CM (2012). Resveratrol enhances the therapeutic effect of temozolomide against malignant glioma in vitro and in vivo by inhibiting autophagy. Free Radic Biol Med.

[49] Gatouillat G, Balasse E, Joseph-Pietras D, Morjani H, Madoulet C (2010). Resveratrol induces cell-cycle disruption and apoptosis in chemoresistant B16 melanoma. J Cell Biochem.

[50] Castillo-Pichardo L, Dharmawardhane SF (2012). Grape polyphenols inhibit Akt/mammalian target of rapamycin signaling and potentiate the effects of gefitinib in breast cancer. Nutr Cancer.

[51] Fukui M, Yamabe N, Zhu BT (2010). Resveratrol attenuates the anticancer efficacy of paclitaxel in human breast cancer cells in vitro and in vivo. Eur J Cancer.

[52] Krishan A (1975). Rapid flow cytofluorometric analysis of mammalian cell cycle by propidium iodide staining. J Cell Biol.

